# A double-slit experiment with human subjects

**DOI:** 10.1371/journal.pone.0246526

**Published:** 2021-02-11

**Authors:** John Duffy, Ted Loch-Temzelides

**Affiliations:** 1 Department of Economics, University of California, Irvine, Irvine, California, United States of America; 2 Department of Economics, Rice University, Houston, Texas, United States of America; Bucharest University of Economic Studies, ROMANIA

## Abstract

We study a sequence of “double-slit” experiments designed to perform repeated measurements of an attribute in a large pool of subjects using *Amazon’s Mechanical Turk*. Our findings contrast the prescriptions of decision theory in novel and interesting ways. The response to an identical sequel measurement of the same attribute can be at significant variance with the initial measurement. Furthermore, the response to the sequel measurement depends on whether the initial measurement has taken place. In the absence of the initial measurement, the sequel measurement reveals additional variability, leading to a multimodal frequency distribution which is largely absent if the first measurement has taken place.

## Introduction

A cornerstone of axiomatic modeling of human decision-making holds that individuals have a well-defined ranking over possible deterministic or random alternatives [1–3]. As an example, this assumption implies that each individual has a full preference ranking among, say, the continuum of colors in the visible spectrum. Recent studies argue that it is plausible that the preference ranking (say, between two colors in the spectrum) emerges as part of the measurement, or elicitation process itself [4]. Some experimental evidence provides support for this view [5]. Our investigation is motivated by the influential double-slit experiment, often used to demonstrate wave-like interference in physics. We perform repeated elicitations of the same attribute and study the final pattern resulting from subjects’ responses conditional on whether previous measurements have occurred.

**Fig 1:**
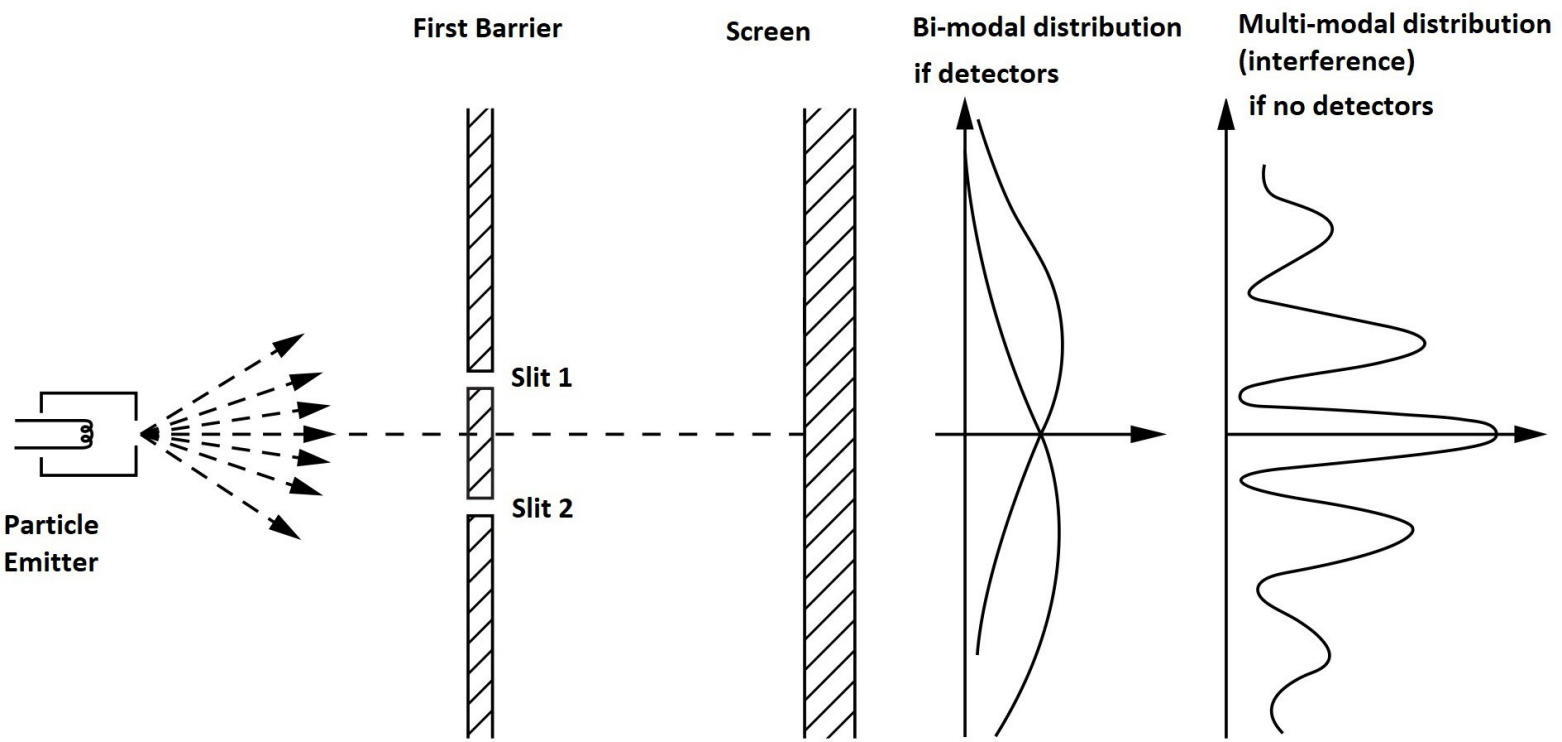
The double slit experiment in physics. Adapted from Feynman, Leighton, and Sands, 1965.

The double-slit experiment has a long tradition as both an idealized thought experiment and an expositional tool [6]. While established in a physics context, the experiment can give rise to an elegant mathematical model via the introduction of non-commutative selective measurement operators [7]. In other words, the mathematical foundation is a logical construction that is independent of its physical interpretation. This modeling, in turn, can be interpreted and used in different contexts, including in the study of human subjects [8–10]. In the idealized physics framework, the double slit experiment involves a subatomic particle emitter firing particles in a general direction. The particles are emitted one at a time and encounter a first barrier, which allows them to pass only through two slits. The final position and overall distribution of those particles which pass through the first barrier is recorded when they hit a second screen as illustrated in Fig 1. The most salient feature of the double-slit experiment in physics concerns the shape of the distribution describing the pattern created on the second screen. This depends on whether the experimenter elicits information about which slit a particle passes through the first barrier. For example, if detectors identify whether particles went through slit 1 or through slit 2 in Fig 1 then a bimodal distribution is observed on the second screen. On the other hand, if there is no detection of which slit the particles passed through, then a multimodal *interference pattern* emerges as also shown in Fig 1 As interference is a property of waves, the experiment reveals that subatomic particles demonstrate a form of “duality;” they may exhibit wave-like properties when not observed, and particle-like properties when they are observed.

Our ultimate goal is to determine if and under what conditions choices made by humans exhibit wave-like properties. Such a distinction would be surprising, but perhaps not more so than the claim, now universally accepted, that matter can exhibit a wave-particle duality. There are some interesting parallels already in place. First, as we mentioned already, it is plausible to think of the elicitation process as “creating” a preference ranking. Second, findings in psychology have highlighted that the order in which response alternatives are presented to respondents may lead to significantly different selections (non-commutativity) [11].

## Materials and methods

We devise and conduct a double-slit experiment with human subjects. While the double-slit experiment is often studied in the context of quantum mechanics, we do not take a stance on whether wave-like human behavior results from quantum mechanical processes in the brain. Interference may potentially play a role in a variety of cognitive processes, and its origin could be entirely classical, for example, resulting from neural oscillators [12, 13].

For our purposes, the double-slit structure provides a straightforward way of creating a bare-bones elicitation process that captures the interaction between subjects and the experimenter during repeated measurements of an attribute. A virtue of our approach is in that it does not rely on the interpretation of “ambiguous concepts,” or any probabilistic assessments from the subjects. We will report on successive measurements of the following type: from a scale 0–10 (in increments of 0.5) state your agreement with the statement *“I like the color green*.” The elicitation of preferences regarding a color seems neutral enough to protect our analysis from any significant “experimenter demand” or Hawthorne effects [14]. Subjects were further instructed that 0.0 means “completely disagree,” 5.0 means “neither agree nor disagree,” and 10.0 means “completely agree.” Following the setup of the physics experiment, we distinguish between two main treatments, each containing two measurements. In both treatments, we ask the above question twice of each subject. What distinguishes the two treatments is the *way* we elicit the attribute value initially. In the first stage of treatment I, the *monitored treatment*, each subject’s individual choice was elicited and recorded by the computer program and reported back to the subject, so that it was very clear that their choice was being recorded. After seeing their first stage choice and clicking on a “next” button, subjects whose first stage response passed through either slit 1 (attribute value in *{2*,*2*.*5*,*3}*) or slit 2 (attribute value in *{7*,*7*.*5*,*8}*) proceeded to stage 2 where they were again asked their opinion of the same statement in stage 2 and where their choice was again recorded. By contrast, in treatment II, the *unmonitored treatment*, we do not elicit the value of subjects’ opinion in the first stage. Instead, we ask subjects to think of their numerical choice and to “keep this number in your memory for a moment”. Next we ask whether a subject’s attribute choice belongs to the set {2, 2.5, 3, 7, 7.5, 8} which comprises the union of the two slits: in *{2*,*2*.*5*,*3}∪ {7*,*7*.*5*,*8}*. Subjects simply answer Yes or No to this question. Subjects who answered Yes to this question immediately proceeded to stage 2 where they were again asked their opinion of the same statement in stage 2 but this time their choice was recorded just as in stage 2 of the monitored treatment. In both treatments, subjects who did not pass through the slit in stage 1 were immediately sent to complete a brief demographic survey and paid. Subjects who moved on to stage 2 completed that stage before moving on to the same demographic survey. Notice that there is no mention of slits in either treatment. Importantly, while we know something about subjects’ first stage attribute choice in the unmonitored treatment, we do not know the precise value of their first stage choice or which slit it passed through. Using this design, subjects should understand that their first-stage choice was not known to the experimenters in the unmonitored treatment while it was known to the experimenters in the monitored treatment.

### Ethics statement

Prior to participating, subjects were asked to read a statement about the study that did not reveal the aims. They were told that it could take up to 10 minutes to complete and if completed they would earn USD $0.50. They had to give consent to our recording their data and were given contact information for the UCI Institutional Review Board if they had any grievances. By clicking Accept on this first page consent agreement, they proceeded on to the main task.

## Results and discussion

The details of the experiment together with some robustness results from a follow-up experiment are provided in the S1 File. We studied responses from a total of 1,620 subjects, recruited from *Amazon’s Mechanical Turk*. Following completion of the questionnaire, subjects were paid for their participation (USD$0.50 per subject).

Subjects were randomly assigned to either the monitored (n = 808) or to the unmonitored (n = 812) treatments. A total of 702 subjects passed through the two slits to the second measurement stage, most of them from the *{7*,*7*.*5*,*8}* slit, indicating an overall favorable view of the color green. Subjects did not receive any additional information between the two stages. Decision theory puts no restrictions on the predicted agreement with the statement *“I like the color green*,*”* as some subjects might like green more than others. However, as no information or payoff-relevant characteristics are involved in this elicitation, standard theory predicts that the second stage answer should be identical to the first, regardless of whether the first stage answer was monitored (subjects’ first stage choice was recorded and reported back to them) or unmonitored (subjects’ first stage choice was not observed or recorded). Our results are at significant variance with this prediction.

**Fig 2:**
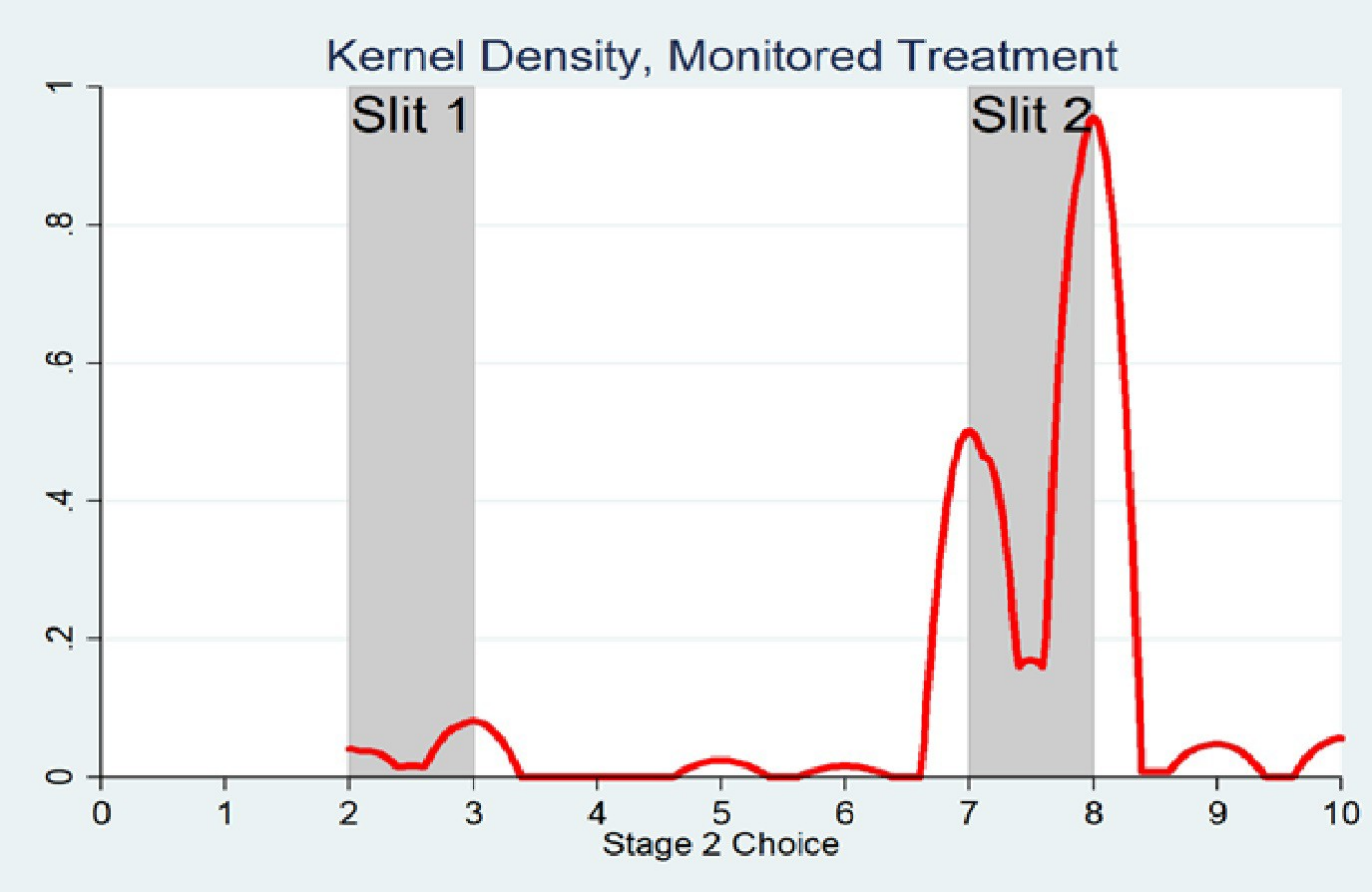
Estimated kernel density, monitored treatment.

**Fig 3:**
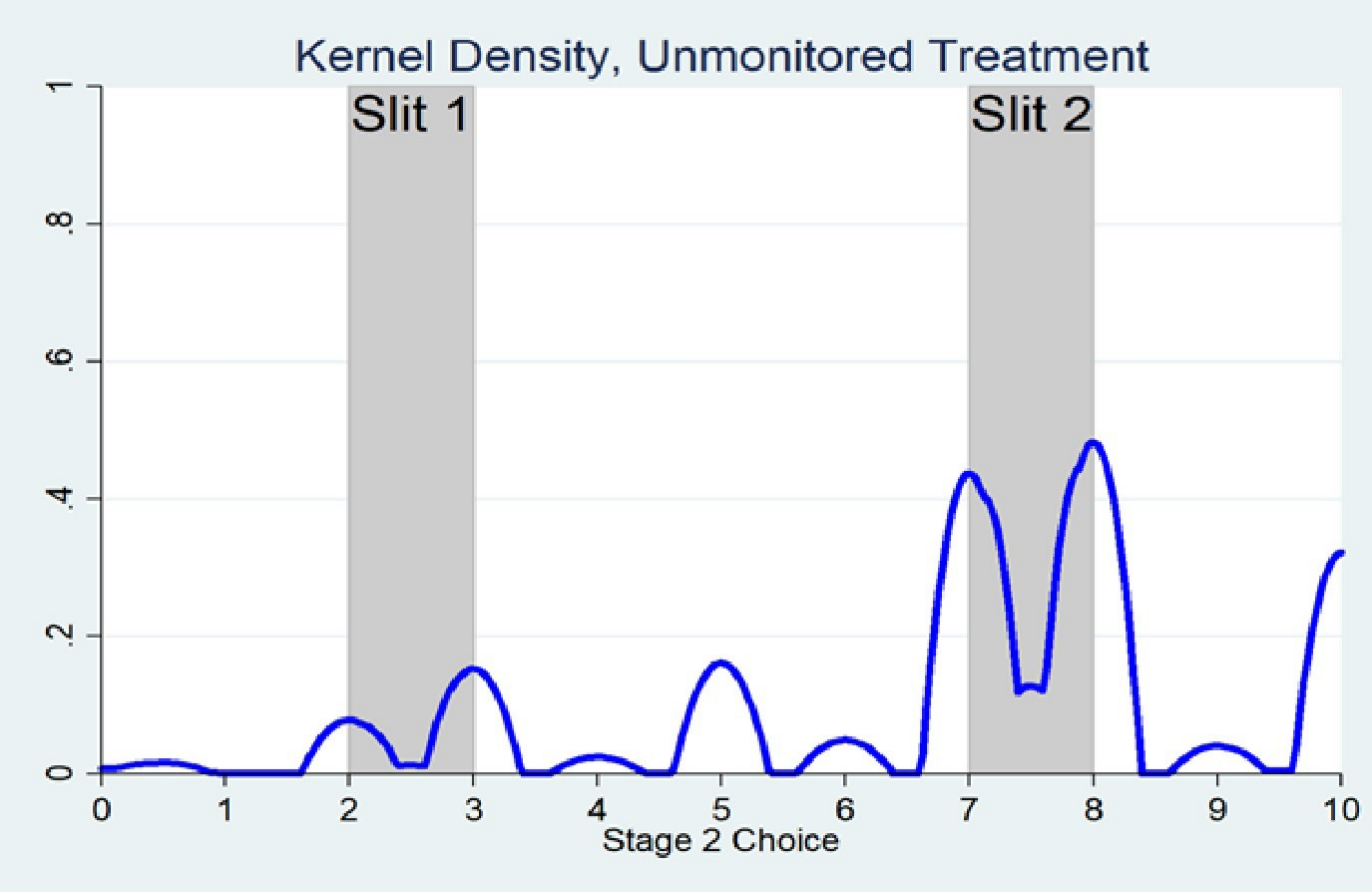
Estimated kernel density, unmonitored treatment.

Figs 2 and 3 show kernel density plots of second stage choices (by those passing through the slits) in the Monitored and Unmonitored treatments respectively. These were estimated in Stata using the Epanechnikov kernel function, but other kernel functions give similar results.

Notice that the estimated kernel density for the Unmonitored treatment is more diffused than for the Monitored treatment and the former covers the entire range of possible 2^nd^ stage guesses, 0–10, whereas the estimated kernel density for the monitored treatment is more concentrated around the slit choices between 7–8 and does not cover the entire range of possible 2^nd^ stage guesses, beginning only at around 1.5. The non-monotonicity of the density *within* each slit is present in both treatments and indicates a preference to report an integer value, as opposed to a decimal.

Confirming Figs 2 and 3, we find that the distributions of second-stage responses made by those passing through the slits in the Monitored and Unmonitored treatments are significantly different from one-another according to a Kolmogorov-Smirnov test, p < .0001 (see also A5 Fig in S1 File in the Methods section). Specifically, second-stage guesses are more dispersed over the admissible range in the Unmonitored treatment as compared with the Monitored treatment. Similarly, we also find a large difference in the dispersion of second stage responses between the two treatments, as measured by their standard deviations: the standard deviation in the Unmonitored treatment, 2.32, is more than 1.5 times greater than the standard deviation in the Monitored treatment, 1.49. This difference is significant according to an F-Test for equality of two standard deviations p < .0001. Finally, although an approximately equal number of subjects were randomly assigned to each treatment, 237 subjects passed to the second stage in the Monitored case, while 465 passed to the second stage in the Unmonitored one. This difference in proportions is statistically significant according to Pearson’s Chi-Squared Test, p < .0001.

To assess the robustness of our results, we modified our experimental design in two ways and we collected additional data. First, we eliminated the 0.5 increments in the Likert scale for responses to the question “I like the color green”. Subjects were instead instructed to choose “whole numbers” 0,1,2,…,10 where 0 means "Completely Disagree", 5 means "Neither Disagree nor Agree", and 10 means "Completely Agree". Second, in the monitored treatment, following the first round choice, we reported back to subjects the number they had chosen in the first round and on the same screen reporting their prior choice we asked them: “Is the number you chose in the set [2, 3, 7, 8]?” In this new version, subjects who answered yes to this question also passed through the two slits to the second round choice in the monitored treatment as well. For this new version of the experiment, we collected responses from 603 participants who did not participate in the original study. We again observed that both the first and second stage choices were skewed to the right, with medians that are greater than the means, indicating that a majority of subjects have a generally favorable opinion of the color green. As in the original design, we found that choices were more dispersed in the Unmonitored treatment as compared with the Monitored one. We again found a statistically significant difference (p < .01) in the standard deviation of stage 2 choices between treatments: the standard deviation in the Unmonitored treatment was about 1.3 times greater than the standard deviation in the Monitored treatment. Second stage choices were more widely dispersed in the Unmonitored treatment as compared with the Monitored one. Although there are some differences in results using this new experimental design partly due to the smaller sample size, we conclude that the restriction of the choice set to integers and making the two treatments more comparable in terms of passage through the two slits does not appear to lead to qualitative differences in our findings. The full robustness analysis can be found in the S1 File.

## Conclusions

Some alternative explanations for our experimental findings are worth considering. One explanation could be related to memory. Subjects, for example, might forget their response between the two measurement rounds. We think that memory-related explanations of our findings are implausible. Most subjects completed the experiment in a few seconds, which is a rather limited time to forget a single-digit number. In addition, to the extent that young subjects have a better memory than older ones, we did not find a statistically significant difference in responses between the two groups. It could be that subjects did not forget, but “changed their mind” and made a new judgement a few seconds after they made their first choice. As no information and no new incentives enter the picture between the two measurements rounds, this explanation would be inconsistent with standard decision theory. Indeed, the only thing that changed is that the first measurement round took place. This measurement-induced change in judgement is exactly what our experiment is designed to capture. In addition, any potential lack of recall of previous answers applies equally to both treatments, and should therefore produce no difference between the Monitored and the Unmonitored cases. Thus, memory issues would not explain why in the Unmonitored treatment the resulting distribution is more bimodal, while in the Unmonitored one it is wavier and multi-modal.

While the finding that differences in feedback from the experimenter may change subjects’ responses is not new, the simplicity of the double-slit sequential measurement of a single attribute allows us to observe exactly *how* the pattern of responses changes from a first-stage measurement. It has not escaped our notice that the unmonitored treatment results in a multi-modal frequency distribution that is reminiscent of an interference pattern. This is in contrast to the monitored case, which results in an essentially bi-modal distribution around the two slits. To the extent that human behavior exhibits wave-like properties, these are not captured by existing models [15]. Our results point to the need for additional research to further investigate this claim. As in the physics experiment, the slit size, the distance between slits, as well as the attribute measured are likely important factors in determining the resulting pattern. While an affirmative conclusion would have profound implications for modeling in decision theory, economics, and related fields, perhaps it would not be more surprising than the analogous conclusion we now know to be true in physics. Modeling the wave-particle duality of human behavior might account for some observed puzzles in human decision-making [16], but may also impose limits on our ability to measure economic attributes with an arbitrary degree of accuracy.

## Supporting information

S1 File (DOCX)

S1 Data (XLSX)

S2 Data (XLSX)
