# Supplementary material for: A double-slit experiment with human subjects
Source: PLoS One. 2021 Feb 11;16(2):e0246526. doi: 10.1371/journal.pone.0246526 (PMC7877746; doi:10.1371/journal.pone.0246526)
Supplement: S1 File — (DOCX) [file pone.0246526.s001.docx]

## **Pre-registration of study design and hypotheses and IRB approval**

The design and hypotheses of this study were pre-registered on the American Economic Review’s registry for randomized controlled trials (RCT) under the title “Measurement and Survey Response,” *RCT ID AEARCTR-0004122*, on March 19, 2019, <https://www.socialscienceregistry.org/trials/4122>. The experimental protocol was pre-approved by the UC Irvine Institutional Review Board, HS#: 2017-3650.

## **Subjects, software, payments and data**

Subjects were recruited from among *Amazon’s Mechanical Turk* workforce residing in the U.S. In total, we recruited 1,846 subjects between April 3, 2019 and February 18, 2020. Subjects were only allowed to participate once. They first read an informed consent study page and if they agreed, proceeded on to the experiment. The experiment was computerized and programmed in *oTree*.^[[1]](#endnote-1)^ Participation required only internet access and took no more than 10 minutes. Subjects who completed the task earned USD$0.50. A total of 1,620 subjects completed the experiment, a completion rate of 87.8%. We report only on the 1,620 subjects who completed the study. The data collected for this study are available on *openICPSR*, at https://www.openicpsr.org/openicpsr/project/120284/version/V4/view

## **Experimental Design**

The experiment consists of two treatments: *Monitored* and *Unmonitored*. Subjects were randomly assigned to one (and only one) treatment. Each treatment consists of one or two stages followed by a questionnaire. In the first stage of both treatments, subjects were asked their opinion of the statement, “I like the color green” on a 0-10 point scale in increments of 0.5, with 0 (10) meaning complete disagreement (agreement) with the statement. Preliminary testing of 12 survey questions with undergraduate students at UC Irvine revealed that this particular question had a more uniform distribution of responses (on a 7-point *Likert scale*), as compared with other survey questions that we considered, though there was a skew in favor of the color green.

In our experiment, whether subjects passed from stage 1 to stage 2 or from stage 1 to the questionnaire depended on whether their first stage answer to the question lied within either of two slits. Slit one was a numerical choice of either 2, 2.5, or 3. Slit 2 was a numerical choice of either 7, 7.5, or 8. If their stage 1 choice was contained within either slit, they passed on to stage 2. Otherwise they transited from stage 1 directly to the questionnaire. Subjects were *not informed* of the procedure for determining whether they passed from stage 1 to stage 2 or directly to the questionnaire in either treatment.

### **Monitored Treatment**

In the Monitored treatment, subjects began stage 1 with the following question:

*Question: Consider you opinion of this statement:*

***I like the color green***

*Think about a number that most closely matches your opinion of the statement above, on a scale of 0 to 10 inclusive in increments of 0.5 where 0.0 means “Completely Disagree” 5.0 means “Neither Disagree nor Agree and 10.0 means Completely Agree.*

*Your opinion*

*[Input box]*

*[Next button]*

After entering their choice and clicking on a Next button, subjects saw a screen recording their choice:

*You were asked to consider your opinion of this statement on a scale from 0 to 10 inclusive in increments of 0.5, where 0.0 means "Completely Disagree", 5.0 means "Neither Disagree nor Agree", and 10.0 means "Completely Agree".*

***I like the color green.***

*You responded: x.x*

*[Next button]*

On this confirmation screen, x.x was the number between 0.0 and 10.0 inclusive corresponding to their first stage choice. This message made it clear to subjects that their first stage choice in the monitored treatment was being recorded by the computer program.

If their choice in the first stage ***did not*** lie in (pass through) one of the two slit intervals, {2, 2.5, 3} or {7, 7.5, 8}, then, after clicking the next button they were immediately sent to complete the ex-post demographic survey.

If their choice in the first stage ***did*** pass through one of the two slits, {2, 2.5, 3} or {7, 7.5, 8} then, after clicking the next button they moved on to the stage 2 screen where they saw this question:

*Question: You are again asked your opinion of the statement:*

***I like the color green.***

*Please enter the number that best corresponds to your opinion of this statement, on a scale from 0 to 10 inclusive in increments of 0.5, where 0.0 means "Completely Disagree", 5.0 means "Neither Disagree nor Agree", and 10.0 means "Completely Agree"*

*Your opinion.*

*[Input Box]*

*[Next button]*

Once they entered a choice and clicked on the next button, their answer was recorded and they immediately moved on to the ex-post survey.

### **Unmonitored Treatment**

In the Unmonitored Treatment, subjects begin stage 1 with the following question:

*Question: Consider you opinion of this statement:*

***I like the color green***

*Think about a number that most closely matches your opinion of the statement above, on a scale of 0 to 10 inclusive in increments of 0.5 where 0.0 means “Completely Disagree” 5.0 means “Neither Disagree nor Agree and 10.0 means Completely Agree. Keep this number in your memory for a moment.*

*[Next button]*

After clicking on the next button, they moved to a screen that posed this question:

*Question: You were asked to consider your opinion of the statement*

***I like the color green***

*on a scale from 0 to 10 inclusive in increments of 0.5 where 0.0 means “Completely Disagree” 5.0 means “Neither Disagree nor Agree and 10.0 means “Completely Agree”*

*Is the number you chose in the set {2.0, 2.5, 3.0, 7.0, 7.5, 8.0}?*

*[Yes button] [No button]*

If they clicked on No, they were immediately sent to complete the ex-post demographic survey. If they clicked on Yes, they moved on to the stage 2 screen where they saw this question:

*Question: You are again asked your opinion of the statement:*

***I like the color green.***

*Please enter the number that best corresponds to your opinion of this statement, on a scale from 0 to 10 inclusive in increments of 0.5, where 0.0 means "Completely Disagree", 5.0 means "Neither Disagree nor Agree", and 10.0 means "Completely Agree"*

*Your opinion.*

*[Input Box]*

*[Next button].*

Once they entered a choice and clicked on the next button, their answer was recorded and they immediately moved on to the ex-post survey.

The ex-post survey asked subjects their country of citizenship, their postal code, their age, their gender and their race; the latter had a drop-down box.

Notice that the primary difference between the monitored and unmonitored treatment is that subjects’ first stage choice in the monitored treatment *is elicited and recorded* while subjects’ first stage choice in the unmonitored treatment is *not elicited*. While we did ask subjects in the unmonitored treatment whether their first stage choice was in the set {2.0, 2.5, 3.0, 7.0, 7.5, 8.0}, this was only to establish whether or not they would pass through the slits to stage 2. Their answer to this question *does not reveal* any additional information about their stage 1 choice. Subjects in the monitored treatment whose choice was in the same slit set {2.0, 2.5, 3.0, 7.0, 7.5, 8.0} automatically passed through to stage 2 and those whose choices were not in this set did not pass through, just as in the unmonitored treatment. Again, subjects were *not informed* of the procedure determining whether they passed from stage 1 to 2.

## **Data overview**

We have data from 1,620 participants. The average age is 35.1 years. Males comprise 58.7% and females 41.3% of our subject sample. The breakdown by race is Asian, 8.99%; Black or African American, 9.77%; Hispanic or Latino, 6.18%; Mixed race, 3.21%; Pacific Islander, 0.25%; White, 72.59%. It is clear from these data that our sample comprises a broad-based representation of people residing in the USA at the time of our study.

Of the 1,620 participants, 812 subjects were randomly assigned to the unmonitored treatment and 808 to the monitored treatment. Of the 812 subjects in the unmonitored treatment, 465 pass through one of the two slits, a pass-through rate of 57.3%. Of the 808 subjects in the monitored treatment, 237 pass through one of the two slits, a pass-through rate of 29.3%. Using Pearson’s Chi-square test of proportions, the null hypothesis of no difference in pass through rates between the two treatments can be rejected p<.0001, in favor of the alternative that pass through rates were higher in the unmonitored treatment. We expect this finding is due to the fact that whether or not subjects pass through the two slits is self-reported in the unmonitored treatment but is explicitly verified in the monitored treatment. The kernel densities of second stage choices shown in Figs 2 and 3 of the text come from the 465 subjects of the unmonitored treatment and the 237 subjects of the monitored treatment, respectively, whose first stage choices were in one of the two slits.

## **Additional results and findings**

In this section, we provide additional results and findings in support of what is reported in the main text of the paper.

### **Monitored treatment**

For the monitored treatment, we have all of the 808 subjects’ first stage choices. Among the subjects in this treatment who passed through either slit, we can compare their first and second stage choices for consistency. Fig A1 shows a histogram of the first stage choices of the 808 subjects in the monitored treatment. Table A1 provides some characteristics of the first and second stage choices in the monitored treatment.

**Figure A1: Histogram of first stage choices, monitored treatment**

**Figure A1: Histogram of first stage choices, monitored treatment**

**Table A1: Stage 1 and 2 Choices in the Monitored Treatment**

| Variable | Stage 1 Choice | Stage1 Choice of Those Passing Through a Slit | Stage 2 Choice |
| --- | --- | --- | --- |
| Mean | 7.438 | 7.179 | 7.342 |
| St. Dev. | 2.504 | 1.475 | 1.491 |
| Coeff. Var. | 0.337 | 0.206 | 0.203 |
| Median | 8 | 8 | 8 |
| Min | 0 | 2 | 2 |
| Max | 10 | 8 | 10 |
| Skewness | -0.953 | -2.566 | -2.128 |
| Kurtosis | 3.531 | 8.591 | 7.966 |
| No. Obs. | 808 | 237 | 237 |

The second column of Table A1 shows properties of all 808 first stage choices in the monitored treatment. Together, Fig A1 and Table A1 reveal that choices in the first stage of the monitored treatment are skewed to the right, with a mean of 7.4 and a median of 8, indicating a majority of participants agree with the statement “I like the color green”. While we do not observe such first stage choices for the unmonitored treatment, since subjects were randomly assigned to the two treatments, we conjecture that the distribution of first stage choices in the unmonitored treatment is similar to that of the monitored treatment.

We next compare the stage 1 choices of participants in the monitored treatment who passed through one of the two slits with their subsequent stage 2 choice. Properties of these 237 observations are shown in the last two columns of Table A2. Notice in particular that there is little difference in the mean, standard deviation and the coefficient of variation (the ratio of the standard deviation to the mean) as well as other measures between the stage 1 decisions of subjects who passed through the slit and their subsequent stage 2 choices. Indeed, the correlation coefficient between stage 1 and stage 2 choices of subjects passing through the two slits in stage 1 is 0.891, which is significantly different from 0, (p<.0001). Of the 237 subjects in the monitored treatment passing through one of the two slits, 202 of those subjects, or 85.2% chose the *exact same number* in stage 2 that they chose in stage 1. Of the remaining 35 subjects (14.8%) who passed through one of the two slits in stage 1 and chose different numbers in stage 2 than they chose in stage 1, 32 subjects adjusted their choice upwards and 3 adjusted their choice downwards in stage 2 relative to stage 1. Due to the latter asymmetry, a Wilcoxon signed rank test for matched pairs indicates that we can reject the null hypothesis that the median deviation is 0 in favor of the alternative that stage 2 choices are higher than stage 1 choices (p<.0001), but this is because the test essentially excludes the 202 out of 237 or 85% of observations where the deviation was exactly 0. The overall mean deviation, i.e., stage 2 choice minus stage 1 choice (among those passing through a slit) is found to be just 0.16 with a standard error of .04. We note that of the 35 subjects who adjusted their stage 1 choice in stage 2, 16 (45.7%) chose numbers in stage 2 that were within the *same slit* that they chose in stage 1, e.g., adjusting from a stage 1 choice of 7 to a stage 2 choice of 8, so that only 19 or 54.3% of the 35 adjusting subjects chose stage 2 numbers that were outside of the slits they passed through in stage 1. Thus overall, 218 (202+16) subjects or 92.0% of all subjects in the monitored treatment who passed through one of the two slits in stage 1 chose numbers in stage 2 that were in the same slit as their stage 1 choice. A further view of the latter finding is presented in Fig A2 which presents an XY bubble plot of stage 1 choices against stage 2 choices of all 237 subjects who passed through the 2 slits in the monitored treatment.

**Figure A2: Stage 1 choices versus Stage 2 choices of those passing through the two slits, Monitored Treatment**


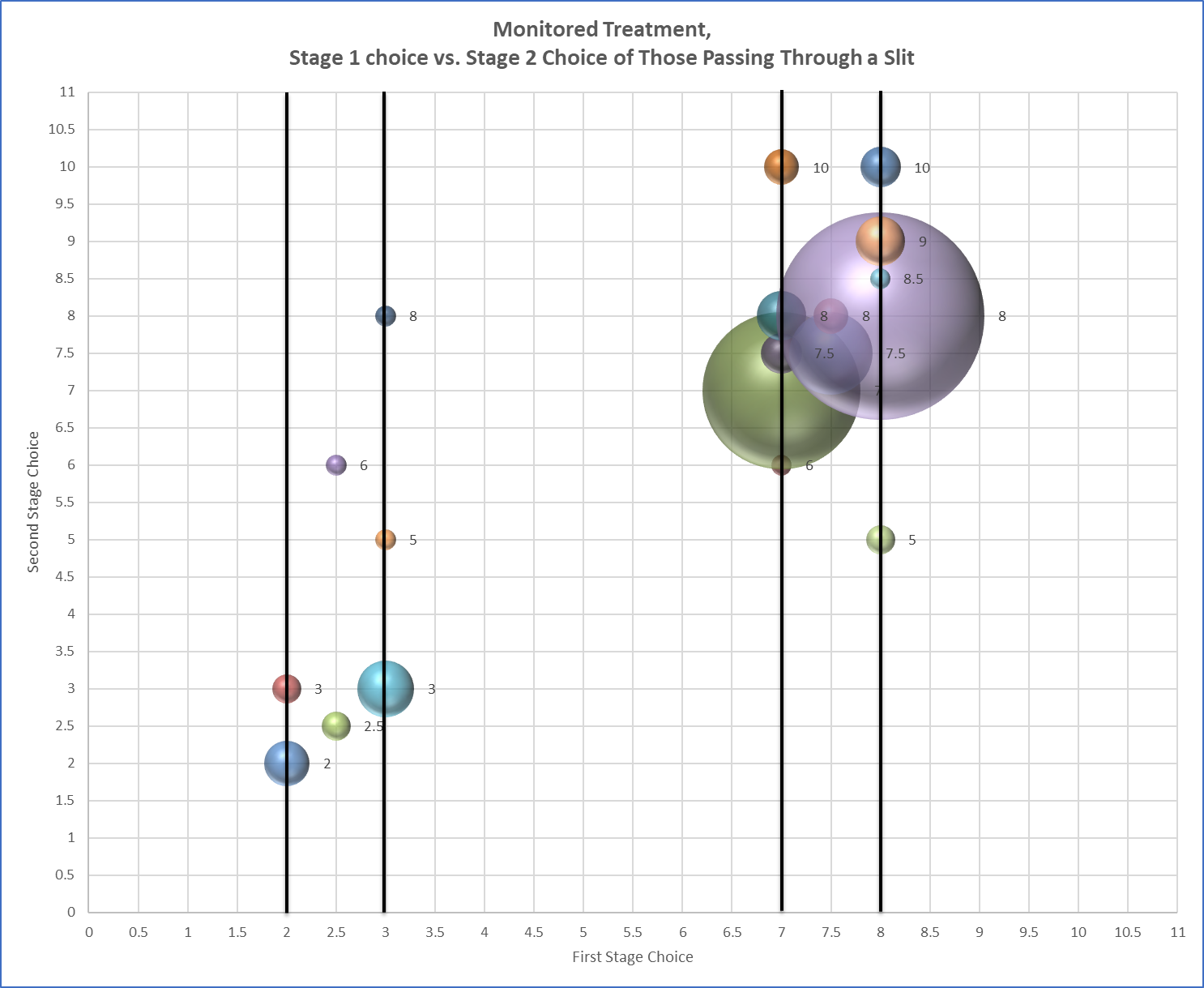


### **Unmonitored treatment**

For the unmonitored treatment, we only have data on second stage choices, conditional on a self-report by the subjects that their first stage guess fell within one of the two slit intervals. As noted earlier, we have 465 such observations. Table A2 provides some characteristics of the stage 2 choices and for comparison purposes, repeats data from Table A2 on stage 2 choices in the monitored treatment.

**Table A2: Stage 2 Choices in the Unmonitored Treatment**

| Variable | Unmonitored Treatment  Stage 2 Choice | Monitored Treatment Stage 2 Choice (From Table A1) |
| --- | --- | --- |
| Mean | 6.967 | 7.342 |
| St. Dev. | 2.316 | 1.491 |
| Coeff. Var. | 0.332 | 0.203 |
| Median | 7.5 | 8 |
| Min | 0 | 2 |
| Max | 10 | 10 |
| Skewness | -0.818 | -2.128 |
| Kurtosis | 3.120 | 7.966 |
| No. Obs. | 465 | 237 |

**Figure A3: Histogram of second stage choices, unmonitored and monitored treatments**


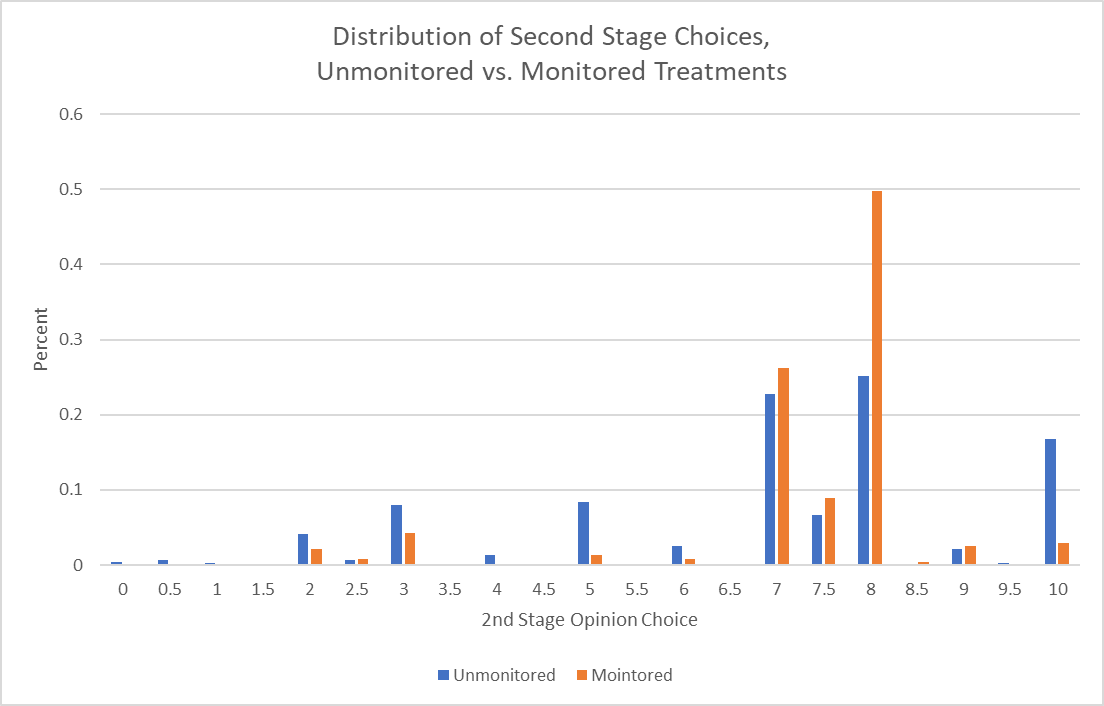


Fig A3 shows a histogram of stage 2 choices for both the unmonitored and monitored treatments. From Table A2 and Fig A3, we see clear evidence that the stage 2 choices in the unmonitored treatment are more dispersed over the admissible 0-10 choice range than are the stage 2 choices in the monitored treatment, which are more concentrated around the two slit intervals of 2-3 and 7-8.

Indeed, an F-test of the null hypothesis that the standard deviations of the second stage choices are equal between the two treatments is rejected (f=0.4143, p<.0001) in favor of the alternative that the standard deviation of stage 2 choices in the unmonitored treatment is greater than the standard deviation of stage 2 choices in the monitored treatment.

Finally, we consider whether the distribution of stage 2 choices in the unmonitored treatment differs from the distribution of stage 2 choices in the monitored treatment. The cumulative frequency distributions of stage 2 choices in both treatments are shown in Fig A4. As this figure reveals, choices in the unmonitored treatment are more widely dispersed than in the monitored treatment, where choices are concentrated around the two slits of 2-3 and 7-8. For this reason, we do not observe first order stochastic dominance of second stage choices in the monitored treatment, but instead, a single sharp crossing point.

**Figure A4: Cumulative frequency distributions of 2^nd^ stage choices, unmonitored and monitored treatments**


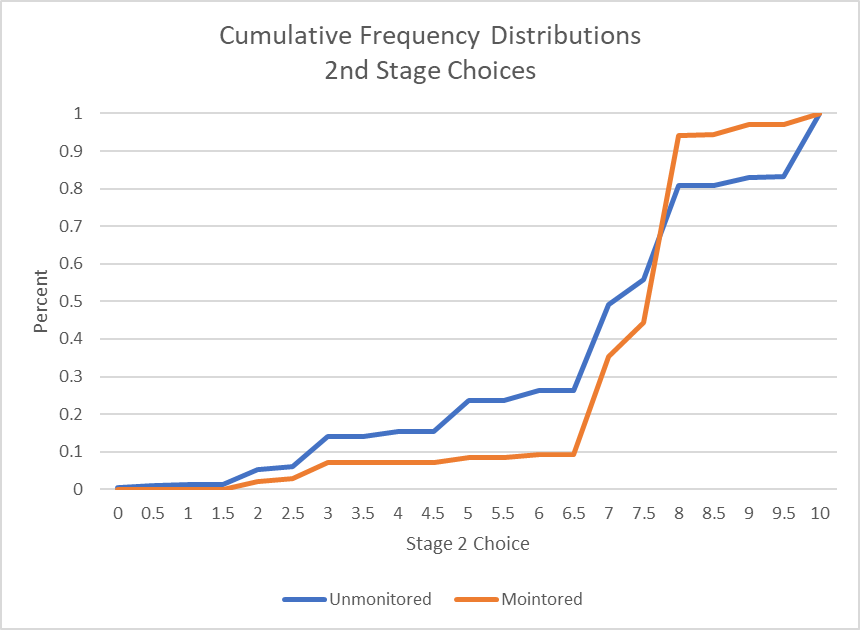


Indeed, a two-sample, two-sided, Kolmogorov-Smirnov test for the equality of distributions functions reveals that we can *strongly reject* the null hypothesis that the distributions are the same, (D= 0.1695, p<0.001). We view the latter test result as providing strong evidence that subjects’ stage 2 choices are greatly affected by whether they are monitored or not. Further evidence that the distributions of stage 2 choices are different between the monitored and unmonitored treatments is presented in the kernel density estimates shown in Figs 2 and 3 of the text.

## **Robustness Checks**

### **Modified Experimental Design**

To assess the robustness of our results, we collected additional data using a modified experimental design of the two treatments reported on in the main text. In this new design, we made two modifications. First, we eliminated the 0.5 increments in the Likert scale for responses to the question “I like the color green”. Subjects were instructed to choose “whole numbers” 0,1,2,…,10 where 0 means "Completely Disagree", 5 means "Neither Disagree nor Agree", and 10 means "Completely Agree". Second, in the monitored treatment, following the first round choice, we reported back to subjects the number they had chosen in the first round and we then asked them: “Is the number you chose in the set [2, 3, 7, 8]?” This is the same question that is asked of subjects in the unmonitored treatment following the first round and subjects who answer yes to this question are allowed to pass through the slits to the second round choice in the unmonitored treatment. In this new version, subjects who answered yes to this question also pass through the two slits to the second round choice in the monitored treatment as well. The idea is to make the questions asked as similar as possible between the two treatments.

For this new version of the experiment, we have complete responses from 603 participants who did not participate in the original study. The original study had 1,620 participants, so this new sample is just 37% of the original sample size. Of these new 603 subjects, 308 (51%) were randomly assigned to the unmonitored treatment and 295 (49%) were randomly assigned to the monitored treatment.

Of the 295 subjects assigned to the unmonitored treatment in this new version, 231 (78%) reported passing through the two slits. Of the 308 assigned to the monitored treatment, 182 (59%) reported passing through the two slits. These numbers of subjects passing through the two slits are greater than in the original treatment reported on in the main text, where 59.3% and 29.3% of subjects passed through the two slits of the unmonitored and monitored treatments, respectively. However, in the present “whole numbers only” design, there are only 11 possible choices (integers), as opposed to the 21 possible choices of the original experiment. The two slits represented 28.5% of the original choice space whereas in this new design they represent 36.6% of the new choice space, so it may not be so surprising that the numbers of subjects passing through the two slits are greater. Alternatively, one can think of the two slits as being relatively larger in this new design.

In the monitored treatment we have subjects’ first stage choices and we compared these with subject’s own reports as to whether or not they passed through the slit, specifically whether or not their first stage guess was in the set [2,3,7,8]. We find that of the 182 subjects who reported passing through the two slits, only 115 actually did so. As we view these misreports as noise, we eliminate the second stage choices of such subjects, which makes our data more comparable to the original design. Table A3 reports on statistics for the modified experimental design similar to what are reported in Tables A1 and A2.

**Table A3: Stage 1 and 2 Choices in the Modified Design**

| Variable | Monitored Treatment  Stage 1 Choice | Monitored Treatment Stage 2 Choice | Unmonitored Treatment Stage 2 Choice |
| --- | --- | --- | --- |
| Mean | 7.525 | 6.861 | 6.654 |
| St. Dev. | 2.271 | 1.853 | 2.355 |
| Coeff. Var. | 0.302 | 0.270 | 0.354 |
| Median | 8 | 7 | 7 |
| Min | 0 | 0 | 1 |
| Max | 10 | 9 | 10 |
| Skewness | -0.974 | -1.596 | -0.711 |
| Kurtosis | 3.594 | 5.112 | 2.543 |
| No. Obs. | 295 | 115 | 231 |

The numbers in Table A3 are similar to those reported in Tables A1 and A2. For instance, we again observe that both the first and second stage choices are skewed to the right, with medians that are greater than the means, indicating that a majority of subjects have a generally favorable opinion of the color green. As in the original design, we find that choices are more dispersed in the unmonitored treatment as compared with the monitored treatment as Fig A5 reveals. We again find a large difference in the standard deviation of stage 2 choices between treatments: the standard deviation in the Unmonitored treatment, 2.355, is about 1.3 times greater than the standard deviation in the Monitored treatment, 1.853. This difference is statistically significant according to an F-Test for the equality of two standard deviations p<.01.

**Figure A5: Histogram of second stage choices, unmonitored and monitored treatments of Integer Choice Data design**

Fig A6 plots the cumulative distribution of stage 2 choices in the unmonitored and monitored treatments. Similar to Fig A4, Fig A6 suggests that second stage choices are more widely dispersed in the unmonitored treatment as compared with the monitored treatment. In this case, however , a two-sample, two-sided, Kolmogorov-Smirnov test for the equality of distributions functions reveals that we *cannot* reject the null hypothesis that the distributions are the same, (D= 0.1335, p=.13). This inability to reject the null of no differences in distributions between the two treatments of the modified, integer-only design is likely owing to the smaller sample size of this robustness check exercise. Finally, further evidence that the distributions of stage 2 choices are different between the monitored and unmonitored treatments is presented in new kernel density estimates (similar to Figs 2 and 3) as shown in Fig A7. We conclude that the restriction of the choice set to integers and making the monitored treatment more comparable to the unmonitored treatment in terms of passage through the two slits does not appear to lead to any qualitative differences in our findings.

**Figure A6: Cumulative frequency distributions of 2^nd^ stage choices, unmonitored and monitored treatments, integer choice design**

**Figure A7: Estimated Kernel Density, Monitored and Unmonitored Treatments, Integer Choice Data**

### **Demographic Data**

Recall that for the original study we collected demographic data on age, gender, race and location. In a second robustness check we ask whether there are any differences in behavior between men and women or between young and old across treatments. For the latter distinction, we computed the median age of our sample, 32, and classified subjects as either young or old depending on whether they were less than or equal to age 32 (young) or older (old). Table A4 reports on our two main demographic variables across the two treatments of our main design (N=1620)

**Table A4: Demographic Statistics, Main Experimental Design**

|  | Monitored N=808 | Unmonitored N=812 |
| --- | --- | --- |
| Percent Female | 44% | 40% |
| Percent Old (Mean Age) | 49% (34.5) | 51%(35.6) |

To examine whether there are differences in behavior by gender and age, we conducted simple, ordinary least squares regression analyses of two variables of interest: (1) whether first stage choices fall within the two slits or not and (2) second stage choices made. We chose these variables as we have data on both variables for each treatment. We examine whether both variables vary with gender and age, with the regression variable “female” being a dummy variable for gender=female and “old” being a dummy variable for age>32. Table A5 reports the results.

**Table A5: First and Second Stage Choices by Gender and Age**

|  | Monitored First Stage Choice in Slits | Unmonitored First Stage Choice in Slits |
| --- | --- | --- |
| Constant | 0.320  (0.025) | 0.629  (0.027) |
| Female | -0.067**  (0.033) | -0.040  (0.036) |
| Old | 0.005  (0.032) | -0.081**  (0.035) |
| Nobs | 808 | 812 |
| $R^{2}$ | 0.01 | 0.01 |
|  | | |
|  | Monitored 2^nd^ Stage Choice | Unmonitored 2^nd^ Stage Choice |
| Constant | 7.473***  (0.149) | 7.121***  (0.162) |
| Female | -0.3305  (0.200) | -0.0733  (0.224) |
| Old | -.001  (0.195) | -0.270  (0.218) |
| Nobs | 237 | 465 |
| $R^{2}$ | 0.01 | 0.01 |

*** Significant at the 1% level; **Significant at the 5% level.

As Table A5 reveals, there are some differences by gender and age in terms of whether first stage choices fall within the two slits, but these are not systematic across the two treatments. Specifically, we see that in the monitored treatment, females’ first round choices are less likely to fall in the two slits relative to males, but this is not the case in the unmonitored treatment. Similarly, in the unmonitored treatment old participants are less likely to report that their first stage choices fell within the two slits relative to young participants, but this is not the case in the monitored treatment. Looking next at the stage 2 choices made by participants passing through the two slits, we do not find any differences by age or gender in these choices. These results are qualitatively the same if we replace the “old” dummy variable by the actual age of the participants in these same regressions. We conclude that our results cannot be explained by systematic differences in the ages or genders of the participants in our study.

1. Chen DL, Schonger M, Wickens C, oTree—An open-source platform for laboratory, online, and field experiments. J Behav. Exp Finance, 9,88-97 2016. [↑](#endnote-ref-1)
